# Supplementary material for: Cognitive function in multiple sclerosis: A long-term look on the bright side
Source: PLoS One. 2019 Aug 29;14(8):e0221784. doi: 10.1371/journal.pone.0221784 (PMC6715181; doi:10.1371/journal.pone.0221784)
Supplement: Attachment — Submitted filename: Achiron.PlosOne.Response to Reviewers.docx [file pone.0221784.s001.docx]

23.6.2019

Dear Editor

Enclosed please find our revised manuscript PONE-D-19-14839 entitled “Cognitive performance in multiple sclerosis: A Long-term look on the bright side”, which we submit after revision after we addressed the points raised during the review process as follows:

**Comment 1. Overall, there needs to be consistencies in terminologies throughout the manuscript. Low cognitive, moderate cognitive, and high cognitive performance or severe cognitive, moderate cognitive, and low cognitive impairments.**

**Specifically, define cognitive resilience in light of the terminology you choose. These will facilitate better comprehension of the manuscript.**

Response: As suggested, we have better defined the cognitive terminologies throughout the manuscript, using the terminology of low cognitive, moderate cognitive, and high cognitive performance.

Accordingly, we have defined cognitive resilience as patients with high cognitive performance after a long-disease duration.

**Comment 2. Results: Page 6, end of the first paragraph: As shown in Table 1, there was also no significant difference in visual functions. This fact needs to be included in the narrative as well.**

Response: We added to the narrative the fact that there was no significant difference in visual function, Results end of first paragraph..

**Comment 3. Figure 1 is exactly the same information as in Table 2. Either one, plus the narrative should be sufficient.**

Response: We omitted Table 2 and added the data to the narrative. Accordingly we changed the order of the figures (Figure 1is now Figure 2, and Figure 2 is now Figure 1), and explained in the narrative the numbers of the inconsistency groups.

**Comment 4. Discussion: Page 7, end of the first paragraph: Can you discuss some of side effects of DMDs?**

Added to the Discussion.

**Comment 5. Page 9: beginning of the second paragraph: If this is a prospective study, how is it possible that data was collected prospectively**?

We mentioned that the study was retrospective cross-sectional, but the data was collected prospectively – as we wanted to highlight the point that patients were evaluated on an on-going basis prospectively. As it seems confusing we omitted the term prospectively.

.

I hope you will find the revised version merit for publication in PlosOne.

With respects

Anat Achiron, MD, PhD

Sheba Medical Center

Tel-Hashomer, 52621, Israel.

Tel: 972-3-5303932; Fax: 972-3-5348186

Email: Anat.Achiron@sheba.health.gov.il
